# Transcriptional co‐activators YAP1–TAZ of Hippo signalling in doxorubicin‐induced cardiomyopathy

**DOI:** 10.1002/ehf2.13756

**Published:** 2021-12-21

**Authors:** Tünde Berecz, Angela Yiu, Orsolya Vittay, Barbara Orsolits, Maxime Mioulane, Cristobal G. dos Remedios, Robin Ketteler, Bela Merkely, Ágota Apáti, Sian E. Harding, Nicola Hellen, Gabor Foldes

**Affiliations:** ^1^ Heart and Vascular Center Semmelweis University 68 Városmajor Street Budapest H1122 Hungary; ^2^ Institute of Enzymology, Research Centre for Natural Sciences Eötvös Loránd Research Network Budapest Hungary; ^3^ Department of Surgery and Cancer Imperial College London London UK; ^4^ National Heart and Lung Institute Imperial College London London UK; ^5^ Victor Chang Cardiac Research Institute Darlinghurst NSW Australia; ^6^ Bosch Institute The University of Sydney Sydney NSW Australia; ^7^ Laboratory for Molecular Cell Biology University College London London UK

**Keywords:** Hippo signalling, YAP/TAZ, Human pluripotent stem cell‐derived cardiomyocytes, Doxorubicin‐induced cardiotoxicity

## Abstract

**Aims:**

Hippo signalling is an evolutionarily conserved pathway that controls organ size by regulating apoptosis, cell proliferation, and stem cell self‐renewal. Recently, the pathway has been shown to exert powerful growth regulatory activity in cardiomyocytes. However, the functional role of this stress‐related and cell death‐related pathway in the human heart and cardiomyocytes is not known. In this study, we investigated the role of the transcriptional co‐activators of Hippo signalling, YAP and TAZ, in human‐induced pluripotent stem cell‐derived cardiomyocytes (hiPSC‐CMs) in response to cardiotoxic agents and investigated the effects of modulating the pathway on cardiomyocyte function and survival.

**Methods and results:**

RNA‐sequencing analysis of human heart samples with doxorubicin‐induced end‐stage heart failure and healthy controls showed that YAP and ERBB2 (HER2) as upstream regulators of differentially expressed genes correlated with doxorubicin treatment. Thus, we tested the effects of doxorubicin on hiPSC‐CMs *in vitro*. Using an automated high‐content screen of 96 clinically relevant antineoplastic and cardiotherapeutic drugs, we showed that doxorubicin induced the highest activation of YAP/TAZ nuclear translocation in both hiPSC‐CMs and control MCF7 breast cancer cells. The overexpression of YAP rescued doxorubicin‐induced cell loss in hiPSC‐CMs by inhibiting apoptosis and inducing proliferation. In contrast, silencing of YAP and TAZ by siRNAs resulted in elevated mitochondrial membrane potential loss in response to doxorubicin. hiPSC‐CM calcium transients did not change in response to YAP/TAZ silencing.

**Conclusions:**

Our results suggest that Hippo signalling is involved in clinical anthracycline‐induced cardiomyopathy. Modelling with hiPSC‐CMs *in vitro* showed similar responses to doxorubicin as adult cardiomyocytes and revealed a potential cardioprotective effect of YAP in doxorubicin‐induced cardiotoxicity.

## Introduction

Traditional chemotherapeutic drug families, like anthracyclines, are highly effective drugs, particularly against breast cancer and haematological malignancies.^1^, ^2^ However, these drugs are the leading cause of cardiac dysfunction in cancer survivors.^3^ Doxorubicin has a multimodal mechanism of inducing cardiotoxicity, including impairment of mitochondrial metabolism, reactive oxygen species production, disruption of Ca^2+^ modulation, and direct DNA damage.^4^ Because the regenerative potential of cardiac cells is known to be low,^5^ controlling cell survival is more important than in other cell types with greater regenerative capacity.

Hippo signalling is an evolutionarily conserved pathway that controls organ size by regulating cell proliferation, apoptosis, and stem cell self‐renewal.^6^, ^7^ The key effectors of the Hippo pathway are the yes‐associated protein (YAP) and transcriptional co‐activator with PDZ‐binding motif (TAZ). These effectors are regulated through phosphorylation, leading to inhibition of nuclear translocation and proteasomal degradation. Dysregulation of the Hippo pathway, via activation of YAP and TAZ, results in uncontrolled proliferation and suppression of apoptosis in adult organs. It is implicated in tumorigenesis of breast, colon, lung, and liver cancers.^8^, ^9^, ^10^ Small‐molecule YAP inhibitors are a potential new therapeutic strategy for various cancers.^8^, ^9^ The Hippo pathway has also been shown to play a crucial role in regulation of cardiomyocytes.^11^ YAP target genes are preferentially expressed in the foetal heart,^12^ and the deletion of YAP in embryonic cardiomyocytes leads to heart hypoplasia.^13^ In adult hearts, YAP silencing led to dilated cardiomyopathy and overexpression of YAP increased cardiomyocyte number and thus heart size in mouse models.^14^ Moreover, when YAP is overexpressed in the adult mouse heart, enhanced preservation of heart function and reduced scar size was observed after myocardial infarction.^13^ Consistent with the *in vivo* observation of doxorubicin‐induced reduction of heart size in mice,^15^ doxorubicin treatment decreased the expression of YAP and caused cell death of neonatal and H9c2 rat cardiomyocytes *in vitro*.^16^, ^17^ In contrast, overexpression of YAP inhibited doxorubicin‐induced cardiomyocyte *in vitro*.^17^ These findings suggest that YAP/TAZ activation is modified in response to doxorubicin treatment and is a promising potential target for regenerative or protective therapy of the heart.^18^ Assessment of YAP/TAZ‐targeted pathway can be used to develop strategies to counter the toxicity of chemotherapies in the human myocardium. However, it is unknown whether chronic manipulation of YAP/TAZ signalling has any potentially deleterious effects on cardiovascular function and homeostasis.

The Hippo pathway and YAP/TAZ transcriptional co‐activators in cardiotoxicity have previously been characterized in H9c2 rat cardiac cells and animal models.^15^, ^18^, ^19^ However, there has been no human‐based study investigating YAP/TAZ transcriptional co‐activators in doxorubicin‐induced cardiotoxicity. In this study, we analysed *ex vivo* human heart samples to evaluate key signalling pathways in doxorubicin‐induced cardiac cell death. RNA‐sequencing analysis revealed that YAP is an upstream regulator of differentially expressed genes between samples from patients with doxorubicin‐induced heart failure and healthy controls. Human pluripotent stem cell (hPSC)‐derived cardiomyocytes are suitable for characterization of molecular mechanisms of cardiotoxicity.^4^, ^20^ We generated a human *in vitro* model for doxorubicin‐induced cardiotoxicity and investigated the role of YAP/TAZ in doxorubicin‐induced cell death. A combination of our *ex vivo* and *in vitro* results suggest that Hippo–YAP/TAZ signalling is involved in anthracycline‐induced cardiomyopathy.

## Methods

### Patient samples

Anonymized human left ventricular myocardial samples were obtained from five patients with doxorubicin‐induced heart failure, undergoing heart transplantation. Healthy left ventricular tissue samples were procured from five non‐failing hearts with no history of cardiac abnormalities, intended for transplantation, but not used by the surgical transplant team of the St Vincent's Hospital Heart and Lung Clinic, Australia. Failing hearts were processed within minutes of the cross‐clamp of the coronary arteries. Non‐failing (donor) hearts were perfused with cardioplegic solution and quickly transported to the Sydney Heart Bank for processing. Approximately 1‐g‐sized pieces of tissue were immediately frozen in liquid nitrogen and stored in nitrogen vapour. Work with human samples protocol was approved by the Sydney Heart Bank Executive and the Human Research Ethics Committee, University of Sydney (HREC Approval 2016/923), and consent was obtained in accordance with these approvals. All work conforms to the principles outlined in the Declaration of Helsinki. Subject characteristics are summarized in *Table* *1*.

**Table 1:** Demographic and clinical data of human heart sample donors

Donor	Patient no.	Sample	Sex	Age	LVEF (%)	Cause of death
2.014	C1	Non‐failing	M	30	>50%	Subarachnoid haemorrhage
3.135	C2	Non‐failing	M	26	>50%	Head injury
6.038	C3	Non‐failing	M	25	>50%	Fractured vertebrae, drowning
6.072	C4	Non‐failing	M	16	>50%	Suicide by hanging
7.054	C5	Non‐failing	M	33	>50%	Chest injury

Donor	Patient no.	Sample	Sex	Age	LVEF (%)	Cause of heart failure
2.064	D1	Doxorubicin	M	13	26	ALL
3.015	D2	Doxorubicin	M	16	N/A	ALL
3.093	D3	Doxorubicin	M	34	17	CLL
4.133	D4	Doxorubicin	M	32	15	Bowel cancer
DOX D	D6	Doxorubicin	M	53	10	N/A

ALL, acute lymphocytic leukaemia; CLL, chronic lymphocytic leukaemia; LVEF, left ventricular ejection fraction.

### Immunohistochemistry

Cryosections of human heart tissues were transferred to glass slides and fixed in 4% paraformaldehyde. Slices were permeabilized with 0.3% Triton X‐100 and blocked with 1% normal donkey serum and 2% bovine serum albumin. YAP/TAZ (1:100) (rabbit, Cell Signaling Technology), caspase‐3 (1:300) (rabbit, Santa Cruz Biotechnology), and troponin I (1:300) (WH0007137M4, Sigma‐Aldrich) primary antibodies were applied in 2% bovine serum albumin overnight. Sections were washed and incubated with secondary antibodies, donkey anti‐rabbit Alexa488 (Jackson IR) and donkey anti‐mouse CF568 (Biotium), each diluted in 1:500 in TBS. Samples were mounted in Fluoromount‐G with DAPI (Invitrogen).

### Haematoxylin and eosin staining

Cryosections of human heart tissues were fixed in 4% paraformaldehyde. After dehydration, the slices were incubated in Mayer's haematoxylin solution (Sigma, St. Louis, MO) for 15 min. Slices were rinsed in running tap water and placed in distilled water. Samples were incubated in aqueous eosin Y solution (Sigma) for 3 min. After dehydration and clearing, slides were mounted.

### Image analysis

Immunofluorescence was analysed using the Nikon A1R laser confocal system; image stacks were obtained with NIS‐Elements AR software. The signal intensity was analysed with ImageJ software. The applied ImageJ macro measured the nuclear signal (colocalization with DAPI) and the cytoplasmic signal around the nuclei in a 5‐px‐wide radius. The difference between these two signals was used as a measure of YAP/TAZ activation. Sarcomere structure and disorganization has been quantitated with the use of the SampEn2D PlugIn in ImageJ software.^21^

### mRNA quantification by reverse transcription–real‐time PCR

Human‐induced pluripotent stem cell‐derived cardiomyocytes (hiPSC‐CMs) were lysed in TriReagent, and total RNA was purified using RNeasy columns (Qiagen) according to the manufacturer's instructions. For controls, total RNA extracted from human foetal and adult hearts was purchased from Takara Bio (Cat Nos. 636583 and 636532).

A total of 500 ng of RNA was subsequently converted into cDNA using High Capacity cDNA Reverse Transcription Kit (Thermo Fisher Scientific). Real‐time quantitative PCR (RT‐qPCR) analyses were performed with TaqMan Gene Expression Assays for YAP (Hs00902712_g1) and TAZ (Hs00794094_m1) (Thermo Fisher Scientific), using GAPDH as the endogenous control. TaqMan Gene Expression Master Mix and a Mastercycler Realplex System (Eppendorf) were used for all RT‐qPCR measurements. mRNA levels were quantified and presented using the 2^−ΔCt^ method. Cell death profiling of hiPSC‐CMs was performed by 96‐well SYBR green‐based quantitative real‐time RT^2^ PCR array with 84 genes (Human Cell Death PathwayFinder, 330231 PAHS‐212ZA, Qiagen).

### RNA sequencing

Sequencing library for RNA sequencing was prepared with TruSeq RNA Library Prep Kit v2 (Illumina), according to the manufacturer's protocols. RNA sequencing was performed using the Illumina HiSeq2500 sequencer with 100 bp paired‐end reads. The quality of the reads was assessed using FastQC Version 0.10.1. The reads were then mapped on the reference human genome, version hg19, obtained from the University of California Santa Cruz (https://genome.ucsc.edu/) using TopHat. For gene‐level analysis, raw counts were produced using HTSeq Version 0.6.1 with human RefSeq annotation and used for differential expression analysis with DESeq2 from Bioconductor (https://www.bioconductor.org/). An outlier identified by principal component analysis was excluded from downstream analysis. Differentially expressed genes between samples from heart failure patients and healthy donors were identified using DESeq2, with age included as a covariant in the model to account for age‐dependent effects. RNA‐sequencing data showed that age had an effect on gene expression profiles in preliminary analysis. Therefore, age was included in the model as a covariate when calculating differential expression with DESeq2 to account for any age‐dependent expression differences. Pathway analyses were performed using Ingenuity Pathway Analysis (IPA, Qiagen, http://www.ingenuity.com/products/ipa). Differential expression values (log_2FC_) were uploaded for the core IPA analyses; activation (positive *z*‐score) or inhibition (negative *z*‐score) of the significantly enriched pathways is shown. For IPA comparison analysis, genes that were up‐regulated and down‐regulated (*z*‐score >1.5 and *z*‐score <−1.5) were analysed. Functional and gene ontology enrichment analysis was performed by interrogating the STRING (https://string‐db.org) and Gene Ontology (GO) databases.

### Preparation of pluripotent stem cell‐derived cardiomyocytes

Human‐induced pluripotent stem cell‐derived cardiomyocytes (iCell Cardiomyocytes, Cat. No. 11713, FujiFilm Cellular Dynamics, Inc.) were thawed and cultured according to the manufacturer's instructions for up to 1 month at 37°C in 5% CO_2_. For optical assays, cells were seeded at 1.8 × 10^4^ viable cells per 0.35 cm^2^ in 0.1% gelatin‐coated glass‐bottom dishes (MatTek, Ashland, MA) to form a synchronous syncytium. For studying the effect of cell density on YAP/TAZ, hiPSC‐CMs were plated on 96‐well plates in densities of 7 × 10^3^ and 2 × 10^4^ cells per well (‘sparse’ and ‘dense’, respectively). YAP and TAZ mRNA levels and YAP/TAZ nuclear translocation were measured after 1 week of culture post‐plating. Replicates were performed with at least three independent cell batches.

### High‐content imaging

ArrayScan High Content Platform (Thermo Fisher Scientific) was used to quantify cell death and YAP/TAZ nuclear translocation. Live cells were stained with TO‐PRO‐3, tetramethylrhodamine methyl ester (TMRM), and caspase 3/7‐FAM as markers of necrosis, mitochondrial membrane potential, and apoptosis, respectively. For cell counting, 2.5 × 10^4^ cells/cm^2^ were seeded in 96‐well plate format. Images were acquired with ×10 objective. Cells were identified with Hoechst fluorescence that defined the nuclear area. Cell loss was calculated from the difference in cell number between control and treated conditions. Data are shown as (% of positive cells − % positive cells in control)∕(100% − % positive cells in control). For nuclear translocation assessment by immunocytochemistry, cells were fixed and stained with YAP/TAZ rabbit mAb (D24E4, Cell Signaling Technology) as the primary antibody and Alexa Fluor 546 anti‐rabbit Ab (Thermo Fisher Scientific) as the secondary antibody, and nuclei were labelled with Hoechst. Nuclear translocation of YAP/TAZ was defined as the differences between their nuclear and cytoplasmic intensities. Experiments were performed on at least three independent cell batches.

### Calcium transients

For functional assessment of the cardiomyocytes, hiPSC‐CMs were cultured as a confluent monolayer (1.8 × 10^4^ cells per well) in 96‐well glass‐bottomed plates (MatTek) coated with fibronectin (Sigma) in a humidified incubator at 37°C. Cells were loaded for 30 min with 4 μM Fura‐4F in serum‐free media (SFM) [phenol‐free DMEM (Gibco, Thermo Fisher Scientific, UK), supplemented with 10 mM galactose, 10 mM sodium pyruvate, and 2 mM l‐glutamine]. Cells were washed with SFM and further incubated for 30 min in SFM prior to experimentation. Calcium transients were recorded using the CellOPTIQ^®^ platform (Clyde Biosciences Ltd, Glasgow, UK).^22^ The Fura‐4F signal was recorded from a 0.2 × 0.2 mm area using a ×40 (NA 0.6) objective lens. Ratiometric imaging was performed using fast switching between light‐emitting diode excitation wavelengths of 355 ± 10 and 380 ± 10 nm. Emitted light was collected by a photomultiplier at 510–560 nm. The two fluorescence signals were digitized at 1 kHz, and the ratio of fluorescence (long wavelength/short wavelength) was used to assess calcium transient properties and beating rates. Specific parameters measured were calcium transient amplitude, time to transient peak (*T*_peak_), time to 50% decay (*T*_50_), 75% decay (*T*_75_), and 90% decay (*T*_90_). Experiments were performed on at least three independent cell batches.

### Toxicity assay screen on 384‐well plates

Cell death and YAP/TAZ nuclear translocation in hiPSC‐CMs in response to 96 antineoplastic and cardiotherapeutic drugs from Prestwick library (www.prestwickchemical.com, 10 μM all) were assessed by high‐content imaging. Experiments were performed on at least three independent cell batches.

### Modification of YAP/TAZ signalling

For overexpression of YAP, the plasmid peGFP‐C3‐hYAP (Addgene plasmid #17843) with *Trans*IT‐LT1 Transfection Reagent (Mirus Bio) was used, according to manufacturer's protocol. Lonza pmaxGFP green fluorescent protein plasmid was used as a GFP control for fluorescence‐based imaging. Successful transfection and overexpression were confirmed using Zeiss LSM‐780 confocal microscopy, Arrayscan high‐content platform, and RT‐qPCR after incubation for 48 h. Gene silencing was achieved with siRNAs for YAP and TAZ (25 nM, SMARTpool: ON‐TARGETplus YAP siRNA L‐012200‐00‐0005; SMARTpool: ON‐TARGETplus WWTR1 siRNA L‐016083‐00‐0005; Dharmacon, Inc.) diluted with 1× buffer; cells were transfected with DharmaFECT™ 1 Transfection Reagent (Dharmacon, Inc.), according to the manufacturer's protocol. Non‐targeting scrambled siRNA control (25 nM; Dharmacon, Inc.) was used as a control. The extent of gene knockdown was quantified after 48 h by RT‐qPCR.

### Statistical methods

Data were plotted in GraphPad Prism Version 8.0 and expressed as mean ± SEM. Unpaired *t*‐tests were used to compare control and doxorubicin‐treated groups of human tissue samples. Paired *t*‐test was used to assess differences between pairs of data of three independent *in vitro* experiments. Comparisons between multiple conditions in three independent *in vitro* experiments were analysed using one‐way ANOVA. Differences at the level of *P* < 0.05 were considered statistically significant, and the following labelling key was used: *P < 0.05, **P < 0.01, ***P < 0.001, and ****P < 0.0001.

## Results

### YAP and ERBB2 are upstream regulators of doxorubicin‐induced differentially expressed genes in human ventricular myocardium samples

**Figure 1:**
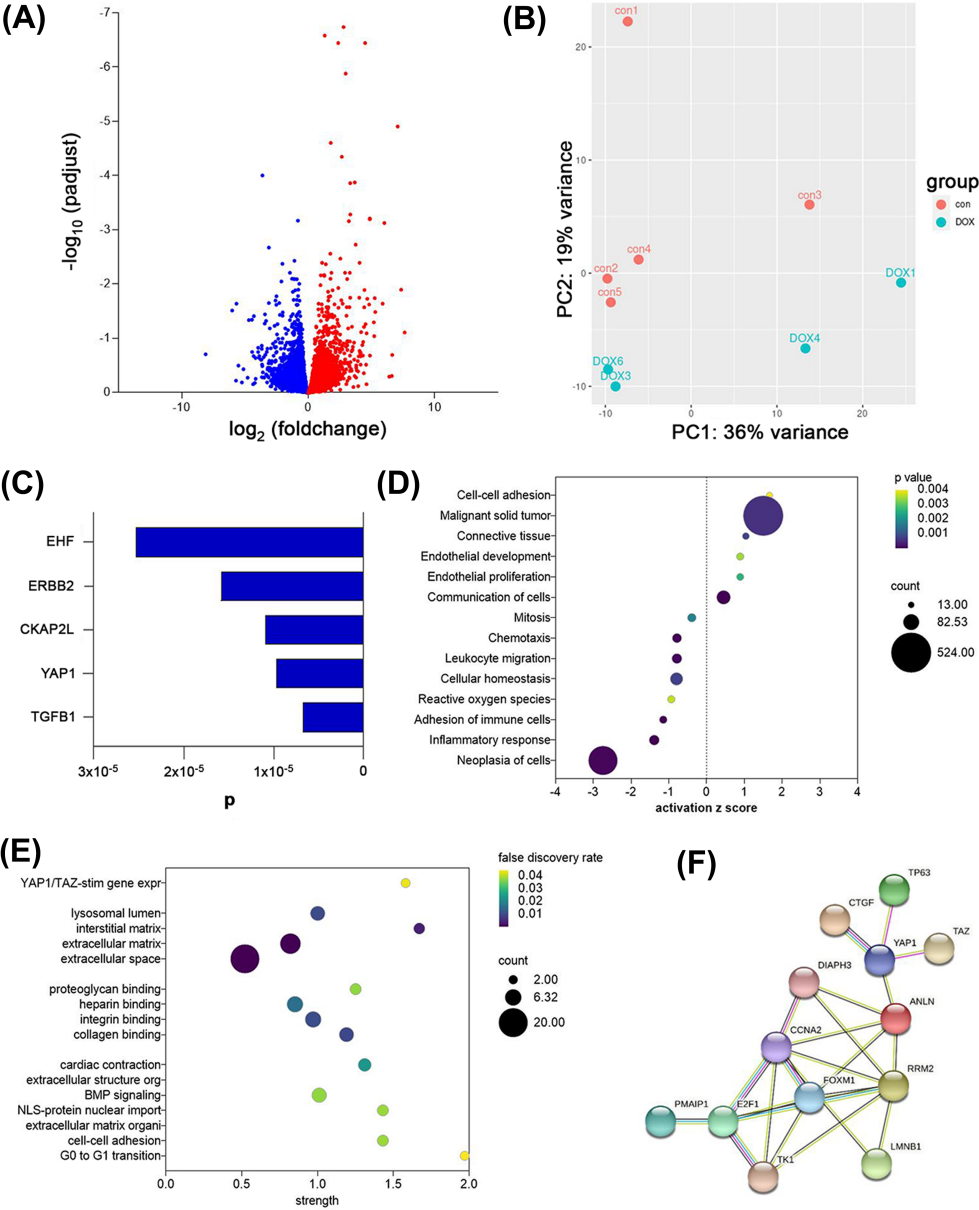
RNAseq‐based transcriptomics profiling of five healthy and five doxorubicin‐induced failing human heart explants. (A) Volcano plot showing differentially expressed genes; up‐regulated genes are shown in red and down‐regulated genes in blue. (B) Principal component analysis shows separation between samples from control group (con) and doxorubicin‐treated group (DOX). The first two principal components of the gene expression dataset are plotted here, and each sample is represented by a dot. (C) Significant upstream regulators identified by Ingenuity Pathway Analysis are shown on the *y*‐axis with the *P* value on the *x*‐axis. (D) An Ingenuity Pathway Analysis showing enriched GO terms as dot plots. The 19 GO processes with the largest gene ratios are plotted in order of gene ratio. The size of the dots represents the number of genes in the significant differentially expressed gene list associated with the GO term, and the colour of the dots represents the adjusted *P* values. IPA activation *z*‐score indicating a predicted activation or inhibition of pathways and functions shown as *x*‐axis. (E) Functional enrichment of network was analysed by STRING‐DB and presented as strength in *x*‐axis. Gene ontology for biological processes, molecular function, cellular components, and reactome pathways are presented. (F) Functional association network diagram showing YAP downstream targets in doxorubicin‐induced failing hearts, generated by STRING‐DB pathway analysis. For network edges, line thickness indicates the strength of data support.

Screening of biopsies has shown that high nuclear translocation and expression of YAP/TAZ is associated with a poor prognosis and reduced survival rate in various cancers.^23^, ^24^, ^25^, ^26^ Following a similar strategy, we performed RNA sequencing on explanted heart samples from patients with doxorubicin‐induced heart failure and control samples from healthy donors (*Table* *1*). Differential expression analysis was performed accounting for age‐related effects. More than 140 significantly differentially expressed genes were identified (*Figure* *1* *A*). Principal component analysis demonstrated a clear separation of control and doxorubicin‐treated samples (*Figure* *1* *B*). IPA identified YAP and ERBB2 as upstream regulators, indicating their putative role in doxorubicin‐induced cardiotoxicity (*Figure* *1* *C*). Interactions, network associations, and gene ontology analysis were analysed by STRING‐DB and IPA and identified active pathways of cell adhesion, remodelling, and extracellular matrix development (*Figure* *1* *D* and *1* *E*). Functional association network also identified differentially expressed YAP downstream targets in doxorubicin‐induced failing hearts, including mitochondrial membrane elements involved in apoptotic signalling, cell cycle transition (regulation of G_0_ to G_1_ phases), and intrinsic apoptotic pathways (*Figure* *1* *F*).

### Doxorubicin‐induced heart failure is associated with the disorganized sarcomere structure and apoptosis

**Figure 2:**
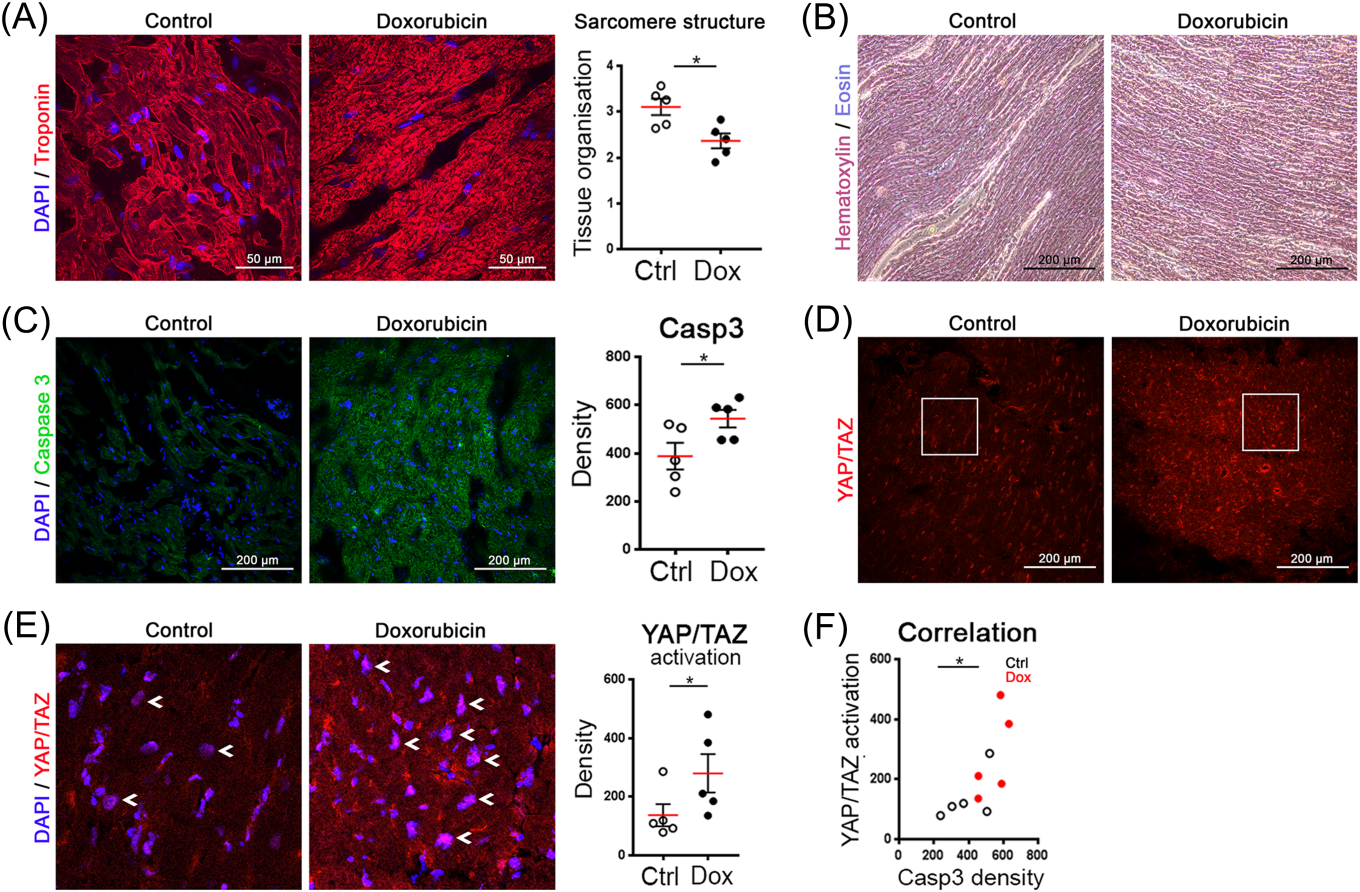
Immunohistochemistry of human heart tissue from healthy and doxorubicin‐treated patients. (A) Doxorubicin treatment causes disorganization of sarcomere structure in cardiac tissue, visualized with troponin I. Representative images (maximum intensity projection of confocal z‐stacks) and analysis of the tissue organization are shown (unpaired *t*‐test, *P < 0.05). (B) Histological examination of left ventricular tissue showing disrupted sarcomere structure after doxorubicin treatment. Haematoxylin and eosin staining. (C) Doxorubicin treatment increases the expression of caspase 3 in the heart tissue. Quantification of cytoplasmic caspase‐3 signal in cardiomyocytes, four regions of interest per heart (unpaired *t*‐test, *n* = 5, *P < 0.05). (D) Doxorubicin treatment increases the expression of YAP/TAZ transcriptional co‐activators in the heart. (E) Sections from (D) images with DAPI. White arrows show activated YAP/TAZ in nuclei (see increased nuclear intensities). Analysis of YAP/TAZ activation in cardiomyocytes, activation = nuclear signal − cytoplasmic signal (unpaired *t*‐test, *P < 0.05). (F) Linear regression analysis between caspase‐3 expression and YAP/TAZ activation (*R* ^2^: 0.5187; *P < 0.05).

To investigate the role of YAP/TAZ transcriptional co‐activators in doxorubicin‐induced heart failure, we compared control and doxorubicin‐treated human heart tissues. Haematoxylin and eosin and troponin I staining revealed disorganization of sarcomere structure in doxorubicin‐treated samples compared with control (*Figure* *2* *A* and *2* *B*). Furthermore, immunostaining showed significantly higher expression of apoptotic marker caspase 3 in doxorubicin‐treated samples compared with control tissue (*Figure* *2* *C*). In addition, we found increased nuclear translocation of activated YAP/TAZ transcriptional factors in doxorubicin‐treated hearts (*Figure* *2* *D*). The presence of YAP/TAZ in cell nuclei is indicative of activation of the transcriptional factors (*Figure* *2* *E*). The linear regression confirmed significant correlation between caspase‐3 expression and YAP/TAZ activation (*Figure* *2* *F*). Collectively, these data indicate the presence of heart failure in the doxorubicin patient hearts and indicate a potential link YAP/TAZ activation and doxorubicin‐induced heart failure.

### Doxorubicin induces YAP/TAZ nuclear translocation and cell death

**Figure 3:**
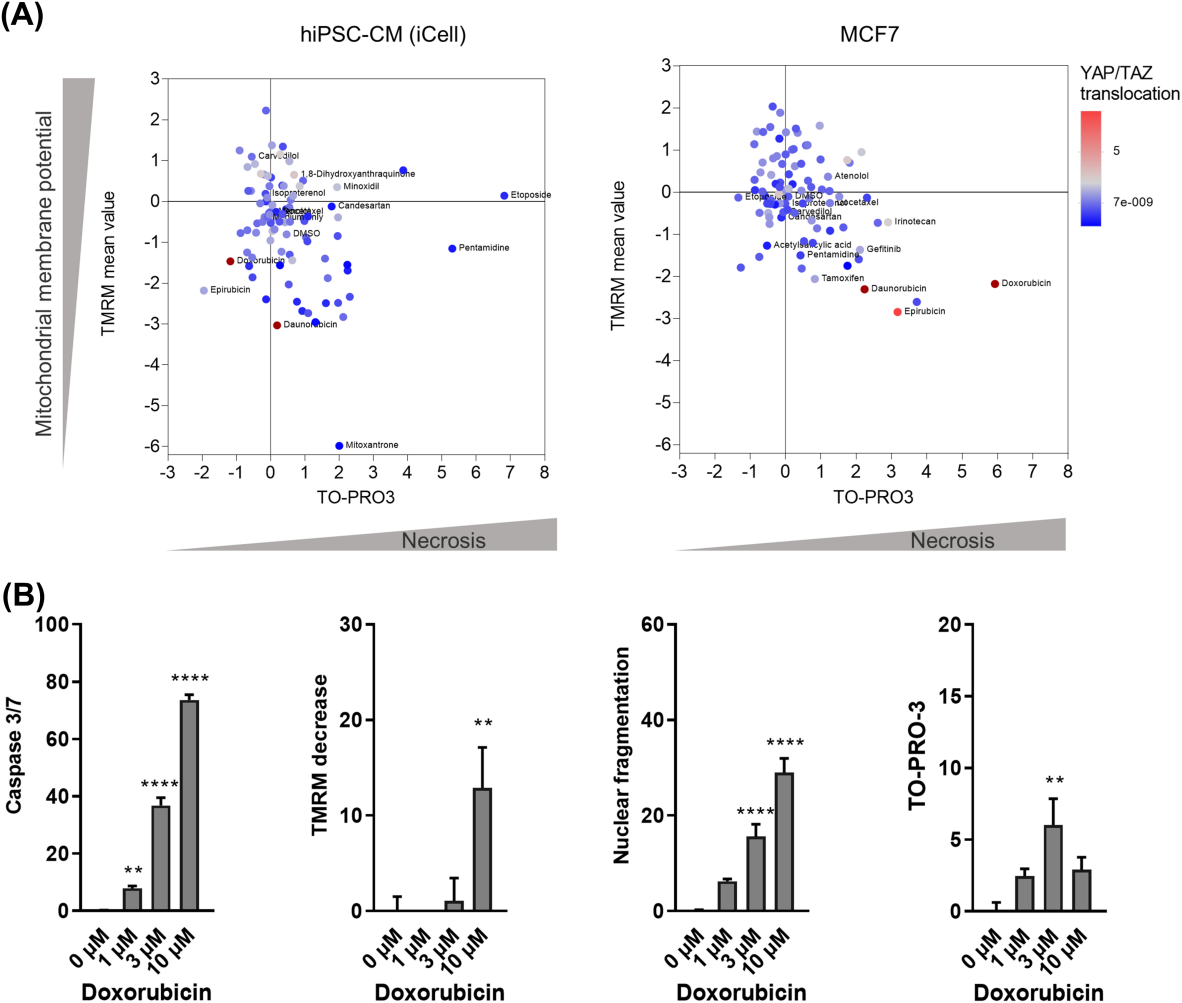
YAP/TAZ activation and cell death profile of hiPSC‐CMs after drug treatment. (A) YAP/TAZ activation is shown colour coded in relation to necrosis as indicated by TO‐PRO‐3 staining and mitochondrial membrane potential (TMRM) levels in response to antineoplastic and cardiotherapeutic drugs in hiPSC‐CMs and MCF7 cancer cells. Each dot represents an individual antineoplastic or cardiotherapeutic drug, *n* = 3 biological replicates. (B) High‐content imaging showing changes in cell death markers in hiPSC‐CMs in response to increasing doxorubicin concentrations, percentage of positive cells is shown on the *y*‐axis (one‐way ANOVA, *n* = 15–18/*N* = 3, *P < 0.05, **P < 0.01, ***P < 0.001, and ****P < 0.0001 vs. control).

Human‐induced pluripotent stem cell‐derived cardiomyocytes (iCell) and an oestrogen and progesterone receptor‐positive human breast cancer cell MCF7 were used for investigating changes in cell death profiles in response to chemotherapeutic drugs. By using the FDA/EMA‐approved Prestwick drug library, we screened 96 antineoplastic and cardiotherapeutic drugs (*Figure* *3* *A*). High‐content/medium‐throughput screening showed that anthracycline agents, such as doxorubicin and daunorubicin, decreased cell viability (as shown by decreased mitochondrial membrane potential) and induced greater nuclear translocation of YAP/TAZ than other classes of drugs in both cell types. A high percentage of cells positive for necrosis marker TO‐PRO‐3 was observed in response to doxorubicin in breast cancer cells but not in hiPSC‐CMs (*Figure* *3* *A*).

Cell death of hiPSC‐CMs in response to doxorubicin treatment was further explored (*Figure* *3* *B*). We found that hallmarks of apoptosis, such as activation of caspase 3/7, mitochondrial depolarization, and nuclear fragmentation,^27^, ^28^ were increased in response to doxorubicin exposure in a concentration‐dependent manner. However, necrosis was only induced in up to 7% of hiPSC‐CMs in response to 3 μM doxorubicin (*Figure* *3* *B*).

### Modulating YAP/TAZ expression and activation in human‐induced pluripotent stem cell‐derived cardiomyocytes

**Figure 4:**
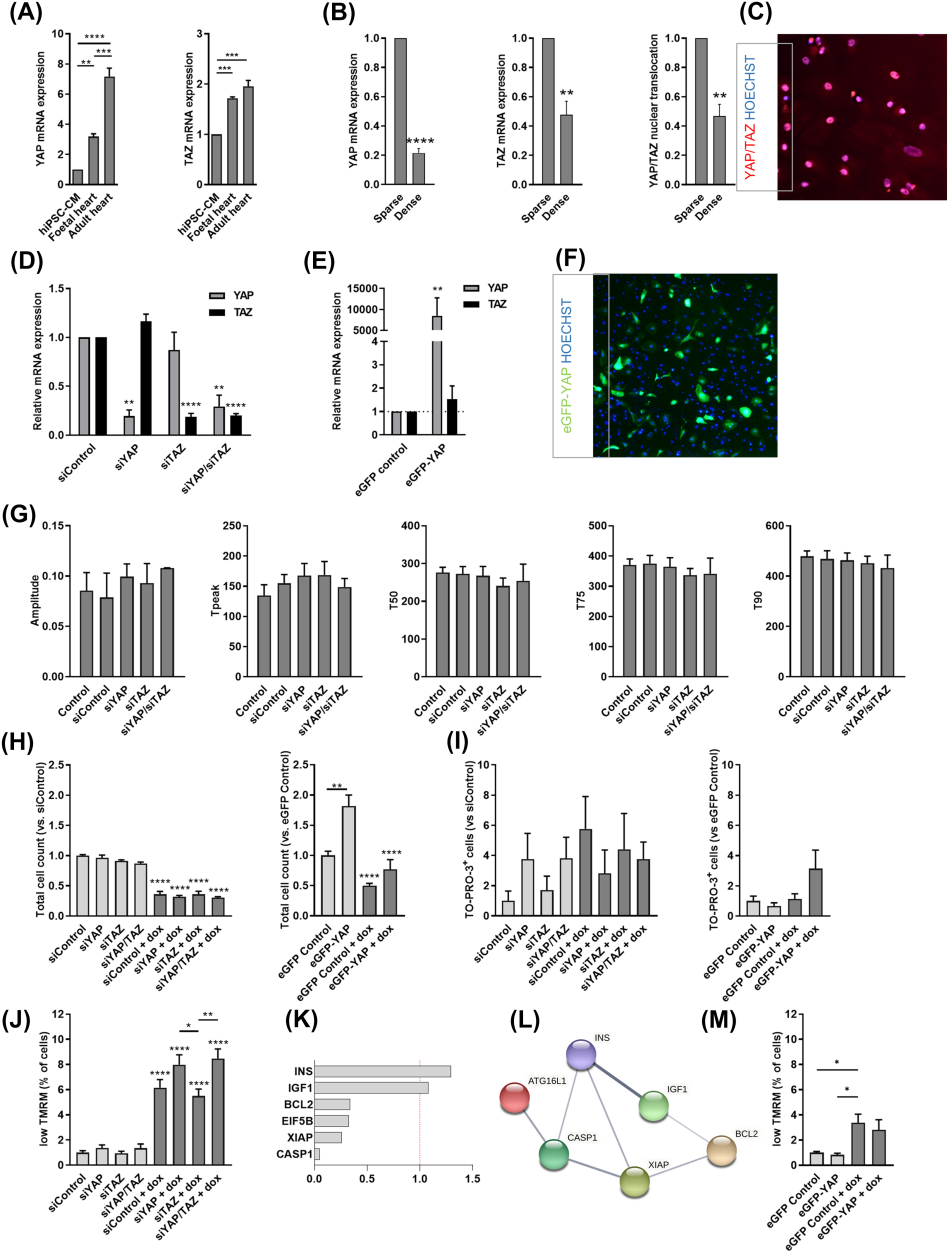
Altered YAP/TAZ expression modulates cell viability and proliferation in hiPSC‐CMs. (A) mRNA levels of YAP and TAZ in hiPSC‐CMs, foetal and adult heart, as assessed by RT‐qPCR (one‐way ANOVA, **P < 0.01, ***P < 0.001, and ****P < 0.0001). (B) Effects of cell density on YAP/TAZ activation and gene expression (paired *t*‐test, **P < 0.01 and ****P < 0.0001). (C) Immunocytochemistry of YAP/TAZ showing nuclear translocation of YAP/TAZ in control hiPSC‐CMs. (D) Silencing of YAP, TAZ, or YAP/TAZ together (one‐way ANOVA, **P < 0.01 and ****P < 0.0001), and (E) overexpression of YAP in hiPSC‐CMs (one‐way ANOVA, **P < 0.01 and ****P < 0.0001). (F) Representative image showing eGFP–YAP expression in eGFP–YAP‐transfected hiPSC‐CMs (paired *t*‐test, **P < 0.01). (G) Calcium transient kinetics in YAP, TAZ, or YAP/TAZ‐silenced hiPSC‐CMs (one‐way ANOVA). Bar charts showing the effects of YAP, TAZ, or YAP/TAZ silencing or YAP overexpression on (H) cell number (one‐way ANOVA, **P < 0.01 and ****P < 0.0001), (I) necrosis (one‐way ANOVA), and (J, M) mitochondrial membrane potential in hiPSC‐CMs in response to doxorubicin treatment (one‐way ANOVA, *n* = 3 biological replicates, *P < 0.05, **P < 0.01, and ****P < 0.0001). (K) Fold changes in mRNA levels of doxorubicin‐activated apoptotic genes in response to YAP siRNA silencing, RT^2^ profiler PCR array. (L) Functional association network diagram showing altered cell death‐related genes in response YAP siRNA in doxorubicin‐treated hiPSC‐CMs, generated by STRING‐DB pathway analysis. For network edges, line thickness indicates the strength of data support.

YAP and TAZ were expressed in hiPSC‐CMs as well as in human foetal heart tissue and adult ventricular cardiomyocytes; however, mRNA levels of YAP and TAZ were lower in *in vitro* differentiated hiPSC‐CMs than those in human *ex vivo* samples (*Figure* *4* *A*). It is known that YAP and TAZ are mechanotransducers, responding to signals such as cell–extracellular matrix contacts and changes in cell–cell contacts.^7^, ^29^ Increases in cell density have been shown to decrease YAP and TAZ activity in other cell populations.^30^, ^31^ To validate our cell culture model, we questioned whether cell density of hiPSC‐CMs can affect YAP/TAZ expression and nuclear translocation. To this end, cells were plated ‘sparse’ and ‘dense’; we found that the levels of YAP and TAZ mRNA were decreased in densely plated cell populations in comparison with sparsely plated populations (*Figure* *4* *B*). Nuclear translocation of YAP/TAZ quantified by automated fluorescent microscopy was also decreased in dense cultures (*Figure* *4* *B* and *4* *C*). These findings corresponded with the known effects of mechanical cues on YAP and TAZ regulated by cell–cell connections.^32^

Our next step was to investigate the effect of transient silencing of YAP and TAZ expression levels on cardiomyocyte function and doxorubicin‐induced cell death. Our results showed that silencing of both YAP and TAZ by siRNA resulted in a decrease in respective mRNA levels (siYAP: 19.6%; siTAZ: 18.6%; and siYAP/siTAZ: 29.3/19.9%) (*Figure* *4* *D*). Next, we transiently overexpressed YAP (by pEGFP‐C3‐hYAP1 plasmid) in hiPSC‐CMs (*Figure* *4* *E* and *4* *F*), which resulted in an over 8000‐fold increase in mRNA levels compared with cells transfected with GFP^+^ plasmid. We first investigated whether YAP/TAZ gene silencing leads to modification of cardiac function by examining calcium transients as a hallmark of cardiomyocyte function. Gene silencing did not significantly affect calcium transient amplitude, time to transient peak, or transient decay (*Figure* *4* *G*). Yet altered sarcomere alignment of hiPSC‐CMs does not exclude altered excitation–contraction coupling and thereby effect on contractile properties in YAP/TAZ siRNA group.

To assess whether the knockdown of YAP and/or TAZ or overexpression of YAP can influence cell survival, total cell count, necrosis marker TO‐PRO‐3, and mitochondrial membrane potential marker TMRM intensity were assessed. High concentration of doxorubicin (15 μM) led to a significant decrease in cell number in hiPSC‐CMs and silencing YAP or TAZ, or YAP/TAZ together did not appear to influence this cellular phenotype (*Figure* *4* *H*). In contrast, YAP overexpression increased the cell count of untreated hiPSC‐CMs and decreased the level of cell loss in response to doxorubicin resulting in comparable total cell count to untreated control cells (*Figure* *4* *H*). When measuring necrosis marker TO‐PRO‐3, we found no significant difference between doxorubicin‐treated and vehicle‐treated cells (*Figure* *4* *I*). On the other hand, decrease in mitochondrial membrane potential was more prominent in response to YAP or YAP/TAZ siRNA compared with TAZ siRNA (*Figure* *4* *J*). These results show that silencing YAP may further induce mitochondrial membrane potential loss after doxorubicin treatment. To confirm this, we used real‐time PCR array to profile the expression of 84 key underlying genes of cellular death: apoptosis, autophagy, and necrosis. We found that YAP siRNA inhibited a set of doxorubicin‐induced cell death genes, including anti‐apoptotic XIAP, Bcl‐2, and eIF5B (*Figure* *4* *K*), which form a network of apoptosis‐related genes (*Figure* *4* *L*). Doxorubicin‐induced mitochondrial membrane potential loss remained non‐significant in YAP overexpressing hiPSC‐CMs (*Figure* *4* *M*).

## Discussion

Traditional chemotherapeutic drug families, like anthracyclines, are highly effective drugs in several cancer types.^1^, ^2^ Improvement in cancer survival rates has increased the recognition of chemotherapy‐induced cardiotoxicity.^3^ These drugs cause cardiac dysfunction leading to a high risk of developing heart failure in cancer survivors.^33^ Yet our current knowledge of the underlying mechanisms and targets involved in drug‐induced cardiotoxicity is limited. The Hippo signalling pathway has been identified in various types of cancer as a growth controlling and tumour suppressor pathway.^34^, ^35^ Based on RNA‐sequencing analysis of left ventricular samples from cancer patients with doxorubicin‐induced heart failure and healthy controls, we found that YAP is one of the top upstream regulators of differentially expressed genes, such as those involved in extracellular matrix rearrangement, oxidative stress, or contractility. As assessed by high‐resolution histology of explants, YAP/TAZ activation in cardiomyocytes was more pronounced in failing than in healthy hearts. This activation was observed parallel with elevated caspase‐3 levels as a marker of apoptosis in cardiomyocytes as well as a loss and remodelling of the whole myocardium. This implicates that YAP may have a role in doxorubicin‐induced cardiotoxicity.

To confirm this, we generated *in vitro* models with hiPSC‐CMs and breast cancer cells and investigated doxorubicin‐induced cell death. Testing a library of 96 antineoplastic and cardiotherapeutic drugs routinely used in clinics, we found that doxorubicin was the strongest inducer of YAP/TAZ nuclear translocation in both breast cancer cells and hiPSC‐CMs. However, cancer cells and hiPSC‐CMs presented with different cell death profiles in response to doxorubicin. Whilst doxorubicin primarily induced necrosis in breast cancer cells, hiPSC‐CMs showed induction of apoptosis but not necrosis. Comparable with the *ex vivo* data, we found that doxorubicin led to elevated levels of caspase 3/7, nuclear fragmentation, and a decrease in mitochondrial membrane potential in a concentration‐dependent manner in hiPSC‐CMs; this indicates that doxorubicin‐induced toxicity occurs primarily through apoptosis.

Similar to in *ex vivo* hearts, doxorubicin induced YAP/TAZ activation in hiPSC‐CMs. YAP/TAZ nuclear translocation (and mRNA levels) was also increased, most likely due to doxorubicin‐induced cell loss and subsequent lower cell density, corresponding with the mechanotransducer role of YAP/TAZ activated by altered extracellular and cell–cell connections.^15^, ^19^ We showed that overexpression of YAP improved doxorubicin‐induced cell death markers such as cell loss and reduction in mitochondrial membrane potential; moreover, YAP overexpression increased cell proliferation, suggesting that increasing YAP expression may be a beneficial strategy against doxorubicin‐induced cardiotoxicity. Given that increased YAP might lead to drug resistance in cancer cells,^36^, ^37^ it is important to consider cardio‐selective overexpression when developing new cardioprotective strategies.^35^, ^38^ Cardiac‐specific YAP activation would selectively protect cardiac cells and allow doxorubicin to simultaneously exert its effect on cancer cells.^39^ In turn, small‐molecule inhibitors of YAP/TAZ are considered potential therapies for various cancers, like mesothelioma and basal cell carcinoma.^8^, ^9^, ^10^ In late preclinical and early phase clinical trials, the targets of these molecules include the MST/LATS of the Hippo core complex direct YAP1 and upstream regulators of the pathway such as Rho GTPases and YES1.^40^ For the cardiovascular system, the Hippo pathway has been identified as a target of pro‐regenerative miRNAs like mir302^41^; thus, a gene therapy approach with AAV vectors using these miRNAs may be a future option in heart failure.

Transient silencing of YAP and TAZ co‐activators resulted in a modest mitochondrial membrane potential loss and a related inhibition of mitochondrial anti‐apoptotic pathways (XIAP, EIF5B, and BCL2). However, as these were not accompanied with significant cell death, the direct role of these inhibitors and thus other Hippo‐dependent elements on cardiomyocyte viability warrants further investigation.

In summary, our results show that doxorubicin‐induced cardiac death is mediated primarily by apoptosis and not necrosis. YAP/TAZ is activated in response to doxorubicin treatment, suggesting that the Hippo pathway plays a role in doxorubicin‐induced cardiotoxicity. Overexpression of YAP rescued doxorubicin‐induced cell loss, by inhibiting apoptosis and through induction of proliferation. Our data add important novel insights into the mechanisms mediating doxorubicin‐induced cell loss and suggest a potential cardioprotective effect of YAP in doxorubicin‐induced cardiotoxicity. Moreover, it further strengthens that hiPSC‐CMs can be used to evaluate patient‐specific drug safety and efficacy, and the underlying molecular mechanisms.^4^, ^20^

## Conflict of interest

The authors declare no competing financial or non‐financial interests.

## Funding

This study was supported by the Medical Research Council (MR/R025002/1 and MC_U12266B), the British Heart Foundation (BHF) Centre of Regenerative Medicine, and the Hungarian National Research, Development and Innovation Fund (NVKP_16‐1‐2016‐0017, NKFI‐6 K128444, and OTKA K 128369).
